# Treadmill Exercise Training Ameliorates Functional and Structural Age-Associated Kidney Changes in Male Albino Rats

**DOI:** 10.1155/2021/1393372

**Published:** 2021-11-30

**Authors:** Heba R. Salem, Manar A. Faried

**Affiliations:** ^**1**^ Medical Physiology Department, Faculty of Medicine, Menoufia University, Shibin El-Kom 32511, Egypt; ^2^Anatomy and Embryology Department, Faculty of Medicine, Menoufia University, Shibin El-Kom 32511, Egypt

## Abstract

Aging is a biological process that impacts multiple organs. Unfortunately, kidney aging affects the quality of life with high mortality rate. So, searching for innovative nonpharmacological modality improving age-associated kidney deterioration is important. This study aimed to throw more light on the beneficial effect of treadmill exercise on the aged kidney. Thirty male albino rats were divided into three groups: young (3-4 months old), sedentary aged (23-24 months old), and exercised aged (23-24 months old, practiced moderate-intensity treadmill exercise 5 days/week for 8 weeks). The results showed marked structural alterations in the aged kidney with concomitant impairment of kidney functions and increase in arterial blood pressure with no significant difference in kidney weight. Also, it revealed that treadmill exercise alleviated theses effects in exercised aged group with reduction of urea and cystatin C. Exercise training significantly decreased glomerulosclerosis index, tubular injury score, and % area of collagen deposition. Treadmill exercise exerted its beneficial role via a significant reduction of C-reactive protein and malondialdehyde and increase in total antioxidant capacity. In addition, exercise training significantly decreased desmin immunoreaction and increased aquaporin-3, vascular endothelial growth factor, and beclin-1 in the aged kidney. This study clarified that treadmill exercise exerted its effects via antioxidant and anti-inflammatory mechanisms, podocyte protection, improving aquaporin-3 and vascular endothelial growth factor expression, and inducing autophagy in the aged kidney. This work provided a new insight into the promising role of aerobic exercise to ameliorate age-associated kidney damage.

## 1. Introduction

The aging process is inevitable and culminates in the functional deterioration of multiple systems [1]. One of the most affected organs by the normal aging process is the kidney [2]. Renal aging has attracted attention because elderly persons are more susceptible to acute kidney injury and chronic kidney disease [3, 4]. Chronic kidney disease is more common as people get older, rising from 4% in people under the age of 40 to 47% in those aged 70 or older [5]. Age-related changes in the kidneys include both anatomical and physiological alterations [6]. These changes impede the kidney's recovery from injury [7]. Even with healthy aging, the number of functioning nephrons declines progressively. Approximately 6200 nephrons are lost per year after the age of 30 [8].

The aged kidney undergoes various structural changes, including glomerulosclerosis, podocyte injury, tubular atrophy, and microvascular changes [9]. Additionally, many aspects of kidney functions are affected, such as glomerular filtration rate, glomerular basement membrane permeability, urine dilution/concentration, acid-base balance, hormonal activity, sodium chloride homeostasis, and control of arterial blood pressure [10].

Aquaporins (AQPs) are a family of transmembrane channels that mainly transport water. Eight AQPs are expressed in the kidney to maintain normal urine concentration. AQP3 is a water-/glycerol-transporting channel that facilitates the transport of water, urea, and glycerol [11]. It also regulates several intracellular signaling processes involved in cell proliferation and apoptosis via permeating glycerol and hydrogen peroxide transport [12].

According to clinical and experimental studies, oxidative stress and inflammation have a significant role in renal aging [13]. Moreover, the aging pathogenesis is aided by the decline in the production of proangiogenic growth factors and cytokines in aged animals, including vascular endothelial growth factor (VEGF), fibroblast growth factor, and transforming growth factor-beta [14].

Autophagy impairment has been implicated in the pathogenesis of many diseases, the development of multiple organ dysfunction, and other negative sequelae [15]. Recently, it was discovered that the dysregulation of autophagy is involved in the pathogenesis of renal aging [16]. Autophagy is a process of cellular recycling involving self-degradation and renewal of protein aggregates and damaged organelles to maintain cellular homeostasis and cell integrity [17]. Beclin-1 is autophagy-related protein involved in the autophagy flux, regulating autophagosome formation [18, 19].

Aerobic exercise has been established to have a renoprotective effect in different experimental models of nephropathy [20, 21]. Moreover, it improves kidney function in patients with chronic kidney disease and enhances renal recovery after acute kidney injury [22, 23]. However, due to limited research, the effect of exercise on healthy renal aging is not well understood.

Thus, this study investigates the effect of treadmill exercise on structural and functional kidney changes in aged albino rats with referral to some underlying mechanisms. To the best of our knowledge, this is the first study to examine the role of exercise on the modulation of desmin (a marker for podocyte injury), VEGF, AQP3, and beclin-1 immunoreactivity in the kidneys of aged albino rats.

## 2. Materials and Methods

### 2.1. Animals

The protocols for animal care and use in this study were approved by the Institution Research Ethics Committee (Institutional Review Board approval number: 11\2021PHYS3. All experimental procedures and animal handling were strictly performed according to the European Committee Directive 86/609/EEC for animal experiments. In this study, thirty male Sprague-Dawley albino rats were used. The animals were housed in standard conditions with a natural light-dark cycle. They were fed standard rat chow and allowed access to water ad libitum. For proper acclimatization, animals were left undisturbed for one week before the start of the experiment.

### 2.2. Experimental Design

Rats were divided into three groups (10 rats per group): (1) young rats (3-4 months old, weighing 150–180 g) were kept sedentary throughout the study period; (2) sedentary aged rats (aged-S: 23-24 months old, weighing 350–400 g) were kept sedentary throughout the study period; (3) exercised aged rats (aged-Ex: 23-24 months old, weighing 350–400 g) performed moderate-intensity treadmill exercise five days/week for eight weeks.

Twenty-four hours after the last exercise session, systolic blood pressure was measured using a rat tail sphygmomanometer (Harvard Apparatus, Ltd., Edenbridge, England) connected to a pneumatic transducer (Harvard Apparatus, UK). Blood pressure changes were recorded via a physiograph (MK–III–S, Narco Bio-Systems, USA). Then, 24-hour urine samples were collected to measure urinary flow and creatinine. After that, fasting retroorbital blood samples were collected, and plasma was separated and stored at −80°C until biochemical analysis was conducted. Finally, all rats were weighed and then were sacrificed by cervical dislocation under anesthesia with 40 mg/kg intraperitoneal phenobarbital injection. The kidneys were immediately dissected, weighed, and preserved in 10% neutral buffered formalin for further histological and immunohistochemical studies.

### 2.3. Exercise Training Protocol

A multilane motor-drive treadmill was used to exercise the rats in the aged-Ex group, five days/week for eight weeks. The treadmill was custom-made as constructed by Rubin and Mickle [24]. Initially, rats were allowed to be familiar with the treadmill for three days before starting the training. On the first day of familiarization, the rats were placed on a static treadmill for 10 minutes. In the second and third days, they ran for 10 min/day at 10 meters (m)/min with no inclination [25]. During the familiarization phase, the rats that did not run on the treadmill were eliminated and replaced with new ones. Then, they were trained for 10 min/day at 10 m/min with no inclination. Over one week, treadmill speed was increased gradually to 20 m/min, maintaining this speed till the end of the study. On the other hand, the duration of the exercise period was progressively increased by 5 min every two days until reaching 60 min/day, which was maintained till the end of the study. The 20 m/min protocol was determined as an aerobic level in rats, corresponding to 50%–70% maximum oxygen consumption, which effectively alleviated early diabetic nephropathy in rats [20]. The sedentary rats stayed on a turned-off treadmill for the same period.

### 2.4. Relative Kidney Weight

The relative kidney weight was calculated using the mean of the right and left kidney weights, corrected for body weight (g/100 g body weight).

### 2.5. Urine Collection

The rats were placed in individual metabolic cages for 24-hour urine sample collection. After measuring urine volumes, urine samples were centrifuged at 1000 revolutions per minute (rpm) for 10 min to remove cells and debris; then, the supernatant was separated and stored at −20°C to determine the creatinine levels.

### 2.6. Blood Sampling

After overnight fasting for 12 hours, blood samples were collected under light anesthesia from the retroorbital venous plexus using heparinized capillary tubes. Then, plasma was separated by centrifugation at 3000 rpm for 15 min and stored at −20°C until biochemical analysis was conducted.

### 2.7. Biochemical Analysis

Creatinine concentrations in plasma and urine and urea concentrations in plasma were determined using colorimetric kits (Spectrum Diagnostics, Egypt), whereas plasma concentrations of cystatin C, interleukin-6 (IL-6), and C-reactive protein (CRP) were determined using the corresponding rat enzyme-linked immunosorbent assay kits (cystatin C: ab201281; IL-6: ab100772; CRP: ab108827, Abcam, Cambridge, UK) according to the manufacturer's instructions. Plasma malondialdehyde (MDA) was determined by thiobarbituric acid-reactive substances and total antioxidant capacity (TAC), representing the cumulative effect of all antioxidants present in plasma were determined using colorimetric kits (Biodiagnostic Company, Dokki, Giza, Egypt) according to the manufacturer's instructions.

### 2.8. Creatinine Clearance

Creatinine clearance was calculated as a ratio of urine creatinine concentration (mg/dL) multiplied by urine volume (mL/min) to plasma creatinine concentration (mg/dL), with values expressed in mL/min [26].

### 2.9. Histological Study

The kidneys were fixed in 10% buffered formalin for 24 h, dehydrated in ascending grades of alcohol (70%–100%), and cleared and embedded in paraffin. Five-micron thick sections were cut by a microtome and subjected to hematoxylin and eosin (H&E) staining for routine histological examination, periodic acid-Schiff (PAS) stain, and Mallory's trichrome stain. Then, the renal cortex was examined under a light microscope.

### 2.10. Semiquantitative Assessment of Tubular Injury Scoring

Using H&E-stained renal sections, we examined at least 20 cortical fields (20 x) in each section for tubular injury scoring based on the percentage of affected tubules as follows: score 0, no tubular injury; score 1, <10% of tubules injured; score 2, 10%–25% of tubules injured; score 3, 26%–50% of tubules injured; score 4, 51%–75% of tubules injured; score 5, >75% of tubules injured at the cortex [27]. The grading percentage was calculated as follows: injury score (%) = (number of injured tubules/number of whole tubules) *x* 100, which was performed by an observer blinded to the groups under study.

### 2.11. Semiquantitative Estimation of Glomerulosclerosis Index

Using PAS-stained renal sections, we utilized a semiquantitative score to evaluate the degree of glomerulosclerosis according to Schaier et al. [28]. The severity of the lesions was examined in 30 randomly selected glomeruli, graded from 0 to 4+ points according to the percentage of glomerular tuft involvement, and scored as follows: 0, 0%; 1+, 1%–25%; 2+, 26%–50%; 3+, 51%–75%; 4+, 76%–100%. The glomerulosclerosis score was calculated as follows: [(1  ×  number of glomeruli with +1) + (2   × number of glomeruli with +2) + (3  ×  number of glomeruli with +3) + (4  ×  number of glomeruli with +4)]/total number of the examined glomeruli, which was performed by an observer blinded to the groups under study.

### 2.12. Immunohistochemical Study

Paraffin sections (5 *μ*m thick) were deparaffinized in xylene for 1–2 min, rehydrated in descending grades of ethanol (100%, 95%, and 70% ethanol) using two changes 5 min each, and rinsed in tap water. To block endogenous peroxidase, they were embedded in 3% H_2_O_2_ for 10 min. Moreover, the sections were treated with 2% trypsin at 37°C for 10 min and then subjected to antigen retrieval. Phosphate-buffered saline (PBS) and 10% normal goat serum (a blocking solution) blocked nonspecific protein binding. The sections were incubated with primary anti-desmin antibody (mouse monoclonal antibody, Abcam, ab8470, dilution 1 : 100), anti-VEGF antibody (rabbit polyclonal antibody, Lab Vision, USA, RB-222-P, dilution 1 : 100), anti-AQP3 antibody (rabbit polyclonal antibody, Abcam, ab125045, dilution 1 : 500), and anti-beclin-1 antibody (rabbit polyclonal antibody, Abcam, ab217179, dilution 1 : 200). The slides were incubated with the diluted primary antibody using PBS for 30 min. After that, a biotinylated goat polyvalent secondary antibody was applied. Drops of streptavidin peroxidase were added to the slide; then, the sections were left for 20 min and washed with PBS for 5 min. Finally, the prepared DAB substrate chromogen (3,3′-diaminobenzidine tetrahydrochloride) was added to the slides. Subsequently, the slides were washed with distilled water. Finally, the sections were counterstained with Harris's hematoxylin.

### 2.13. Quantitative Assessment

Using ImageJ software, version K 1.45, the percentage area of collagen deposition and percentage area of desmin, VEGF, AQP3, and beclin-1 immunoreactivity were measured. For each parameter, ten nonoverlapping fields (40 x) for every specimen were randomly taken using a Leica DML B2/11888111 microscope equipped with a Leica DFC450 camera.

### 2.14. Statistical Analysis

The SPSS version 23 (SPSS Inc., Chicago, IL, USA) was used to analyze the data. Continuous data were expressed as the mean ± standard deviation (SD), whereas the ordinal data (tubular scoring) were expressed as median and range. The significance of differences between groups in all examined parameters was determined by one-way analysis of variance (ANOVA) followed by post hoc Tukey's test, whereas tubular injury scoring was determined by the Kruskal–Wallis test followed by post hoc Mann-Whitney *U* test. *P* values < 0.05 were considered statistically significant.

## 3. Results

### 3.1. Body Weight and Relative Kidney Weight

At the end of the study, aged-S rats showed significantly higher body weight values than young rats (*P* < 0.001). Treadmill training resulted in a significant reduction in body weight compared with that in aged-S rats (*P* < 0.001). An insignificant difference was observed in the relative kidney weight between the experimental groups (*P* > 0.05) (Table 1).

### 3.2. Biochemical Results

Regarding markers of renal function, aged-S rats showed significantly higher plasma creatinine, urea, and cystatin C levels than young rats (*P* < 0.05, *P* < 0.001, and *P* < 0.001, resp.). Treadmill training resulted in a significant reduction in plasma urea and cystatin C levels in the aged-Ex group compared with that in aged-S rats (*P* < 0.05 and *P* < 0.001, resp.), whereas an insignificant difference was observed in plasma creatinine between aged-S and aged-Ex rats (*P* > 0.05). Moreover, compared with the younger rats, aged-S rats showed significantly lower creatinine clearance (*P* < 0.05). An insignificant difference was observed between aged-S and aged-Ex rats in terms of creatinine clearance (*P* > 0.05) (Table 1).

Regarding oxidative stress markers, aged-S rats showed significantly higher plasma MDA and significantly lower total antioxidant capacity than young rats (*P* < 0.001). Treadmill training in the aged-Ex group resulted in a significant reduction in plasma MDA and a significant increase in plasma total antioxidant capacity compared with those in aged-S rats (*P* < 0.05 and *P* < 0.001, resp.) (Table 1).

Regarding inflammatory markers, aged-S rats showed significantly higher plasma IL-6 and CRP levels compared with those in young rats (*P* < 0.05 and *P* < 0.001, resp.). Treadmill training in the aged-Ex group resulted in a significant reduction in plasma CRP (*P* < 0.001) and insignificant change in plasma IL-6 compared with aged-S rats (*P* > 0.05) (Table 1).

### 3.3. Systolic Blood Pressure

**Figure 1:**
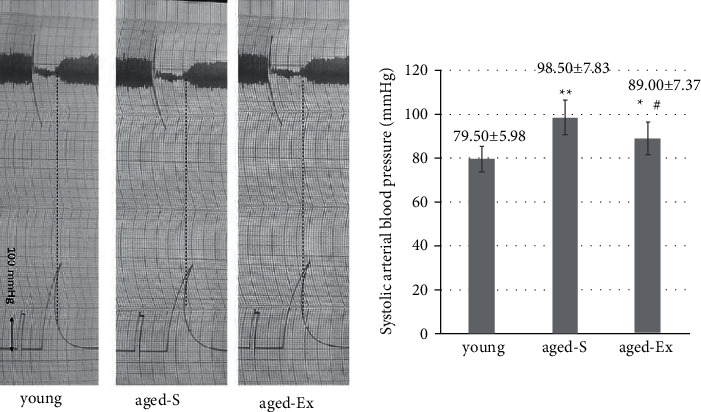
Representative traces and statistical analysis of systolic blood pressure measurements in young, sedentary aged (aged-S), and exercised aged (aged-Ex) rats. ^∗^indicates *P* < 0.05; ^∗∗^indicates *P* < 0.001 compared with the young group; ^#^indicates *P* < 0.05; ^##^indicates *P* < 0.001 compared with the aged-S group. The data are expressed as mean ± SD (*n* = 10).

Aged-S rats showed significantly higher systolic blood pressure than young rats (*P* < 0.001). Treadmill training in the aged-Ex group resulted in a significant reduction in systolic blood pressure values compared with the corresponding values in the aged-S rats (*P* < 0.05) (Figure 1).

### 3.4. Histological Results

#### 3.4.1. Hematoxylin and Eosin (H&E) Results

**Figure 2:**
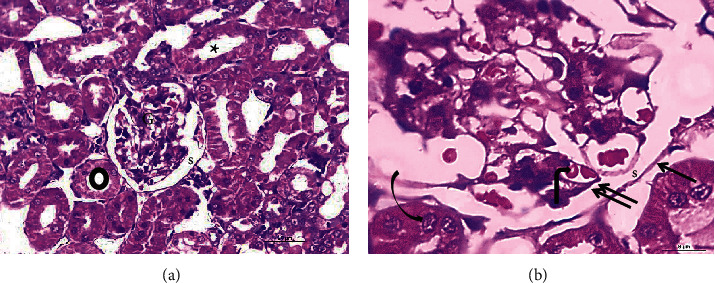
Representative micrographs of H&E-stained sections of the young group (*N* = 10) showing renal corpuscles with a normal glomerular tuft G containing glomerular capillaries (bent arrow). Bowman's space (s) is located between the two layers of Bowman's capsule: parietal layer (arrow) and visceral layer (double arrows). Proximal (asterisk) and distal (circle) convoluted tubules are also seen with normal tubular epithelium (arched arrow). H&E (a): 40x, scale bar = 20 *µ*m; (b): 100x, scale bar = 8 *µ*m. *N* = number of rats/group.

**Figure 3:**
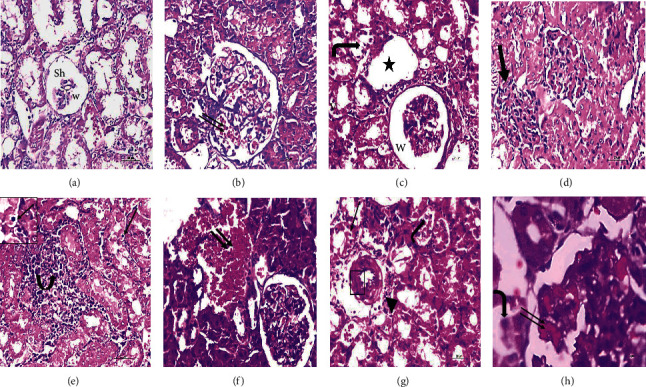
Representative micrographs of H&E-stained renal sections of the sedentary aged group (*N* = 10) revealing marked degenerative changes. Some glomeruli are shrunken (Sh) with a widening of Bowman's space (w); others show obliteration of their Bowman's space (thick arrow). Congested glomerular capillaries (double thin arrows) are also noted. Most renal tubules exhibiting cytoplasmic vacuolation (v), pyknotic nuclei (thin arrows), exfoliating cells (bent arrows), and marked tubular dilatation (star). The renal interstitium showing intense hemorrhage (double thick arrows) and marked inflammatory infiltrate (arched arrow). Thick-walled blood vessels (rectangle) with perivascular inflammatory infiltrate (arrowhead) are also noted. H&E (a–g): 40x, scale bar = 20 *µ*m; (h): 100x, scale bar = 8 *µ*m. *N* = number of rats.

The renal cortex of young rats showed normal renal corpuscles and tubules (Figure 2). Aged-S rats had marked renal degenerative changes. Some renal corpuscles exhibited degenerated glomerular tuft and widening of Bowman's space, whereas others showed absence or narrowing of Bowman's space. Most of the renal tubules had disturbed architecture, displaying exfoliation of their lining epithelium, pyknotic nuclei, vacuolated cytoplasm, and marked dilatation. The interstitial areas showed marked interstitial hemorrhage and intense inflammatory infiltrate. Moreover, thickened blood vessels with perivascular inflammatory infiltrates were observed (Figure 3).

**Figure 4:**
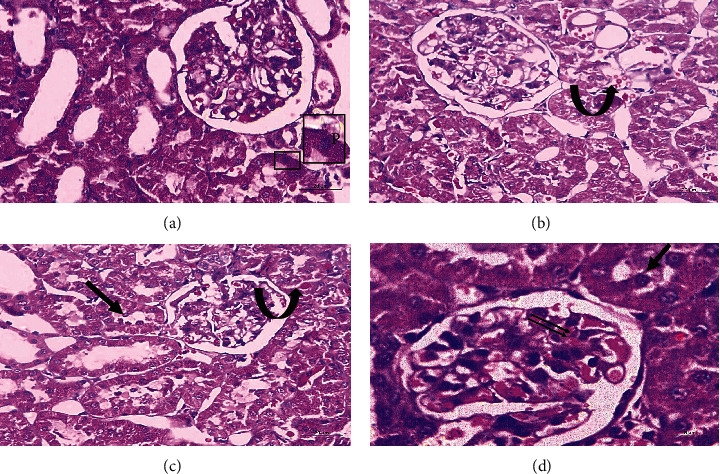
Representative micrographs of H&E-stained renal sections of the exercised aged group (*N* = 10), clarifying amelioration of most of the age-related damaging effects. Slight exfoliation (arrows), few pyknotic nuclei P, and mild interstitial hemorrhage (arched arrows) are noted within some tubules. Few glomeruli show congested capillaries (double arrows). H&E: 40x, scale bar = 20 *µ*m; (d): 100x, scale bar = 8 *µ*m. *N* = number of rats.

On the contrary, treadmill training in the aged-Ex group alleviated most of the age-related damaging effects in the kidney. Slight exfoliation, mild interstitial hemorrhage, and some pyknotic nuclei were still reported in some tubules. In addition, few glomeruli showed congested capillaries (Figure 4).

### 3.5. Semiquantitative Assessment of Tubular Injury Scoring

**Figure 5:**
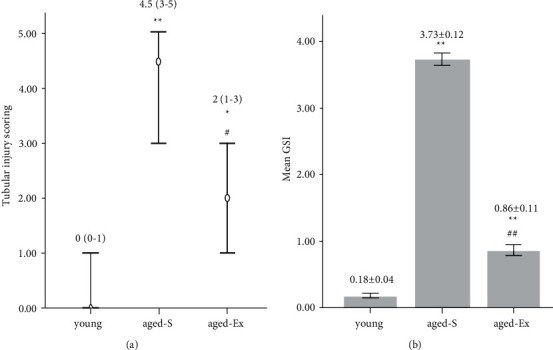
(a) A chart denoting the median and range of tubular injury score in the young, sedentary aged (aged-S), and exercised aged (aged-Ex) groups. ^∗^indicates *P* < 0.05 compared with the young group; ^∗∗^indicates *P* < 0.001 compared with the young group; ^#^indicates *P* < 0.05 compared with the aged-S group. (b) A histogram demonstrating the mean glomerulosclerosis index in the young, aged-S, and aged-Ex groups. ^∗∗^indicates *P* < 0.001 compared with the young group; ^##^indicates *P* < 0.001 compared with the aged-S group. The data expressed as median (range) (a) and mean ± SD (b) (*n* = 10).

A significant increase was observed (*P* < 0.001) in tubular injury score in the aged-S group compared with that in the young ones. Moreover, compared with the aged-S group, the aged-Ex group had a significant decrease (*P* < 0.05) in tubular injury score (Figure 5(a)).

#### 3.5.1. Periodic Acid-Schiff (PAS) and Glomerulosclerosis Index (GSI)

**Figure 6:**
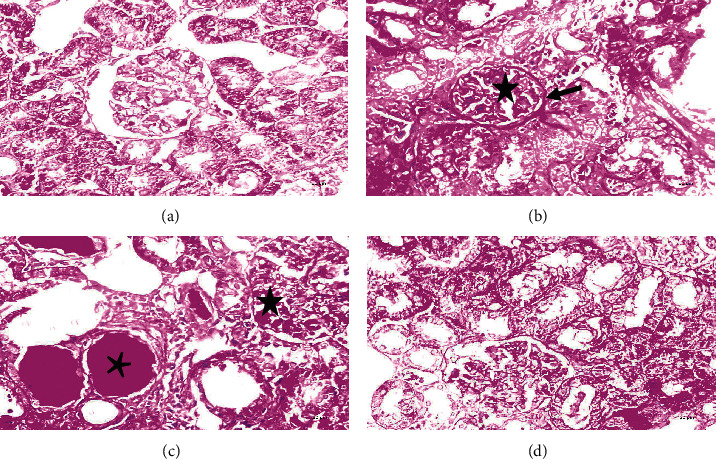
Representative micrographs of PAS-stained renal sections of the different groups under study (a) young; (b, c) sedentary aged (aged-S); (d) exercised aged (aged-Ex); *N* = 10/group. PAS-stained renal sections of the aged-S rats showed deposition of PAS-positive material within the glomerulus tuft (star) with thickening of the glomerular basement membrane (arrow). Deposition of PAS-positive material, mostly cast formation (asterisk), was also noted. These changes are ameliorated in the aged-Ex group (PAS 40x, scale bar = 20 *µ*m). *N* = number of rats.

Compared with the young group, PAS-stained renal sections of the aged-S rats showed deposition of PAS-positive material within the glomerulus tuft (sclerotic changes) with thickening of the glomerular basement membrane. Deposition of PAS-positive material, mostly cast formation, was also noted. These changes were ameliorated in the aged-Ex group (Figure 6).

The degree of glomerulosclerosis was significantly increased (*P* < 0.001) in the GSI in the aged-S group compared with the young group. Compared with the aged-S group, the treadmill exercise significantly lowered GSI in the aged-Ex group (*P* < 0.001) (Figure 5(b)).

### 3.6. Mallory's Trichrome Results

**Figure 7:**
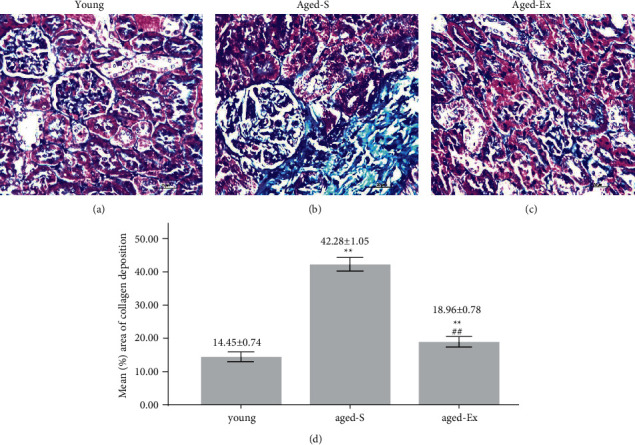
(a–c) Representative micrographs of Mallory's trichrome-stained renal sections of the different studied groups (*N* = 10/group) showing marked fibrosis in the sedentary aged group (aged-S) compared with the control. It is evident that the renal fibrosis is decreased with exercise (40x, scale bar = 20 *µ*m). *N* = number of rats. (d) A histogram denoting the mean % area of collagen deposition in the young, aged-S, and exercised aged (aged-Ex) groups. ^∗∗^indicates *P* < 0.001 compared with the young group; ^##^indicates *P* < 0.001 compared with the aged-S group. The data expressed as mean ± SD.

Mallory's trichrome-stained renal sections revealed intense fibrosis in the aged-S group that was decreased by exercise, indicated by the significant increase (*P* < 0.001) in percentage area of collagen deposition in the aged-S renal sections compared with that in the young rats. In contrast, treadmill training significantly decreased collagen deposition in the aged-Ex group (*P* < 0.001) compared with that in the aged-S group (Figure 7).

### 3.7. Immunohistochemical Results

**Figure 8:**
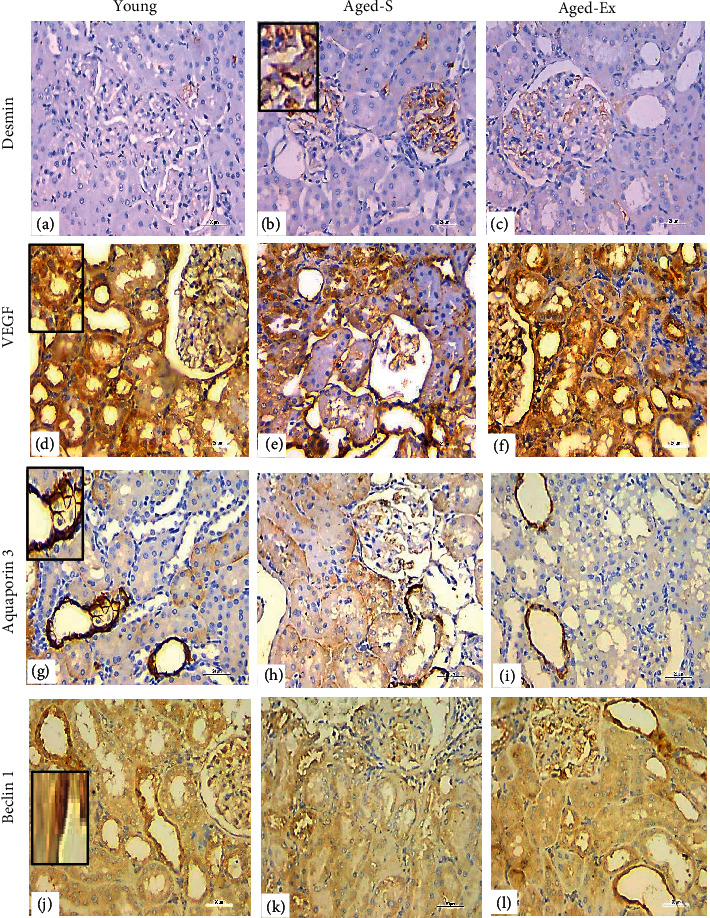
Representative immunostained renal sections of the different groups under study (*N* = 10/group) showing upregulation of desmin (a-b), immunoreactivity with downregulation of VEGF (d-e), aquaporin-3 (g-h), and beclin-1 (j-k) immunoreactivity in the sedentary aged (aged-S) group compared with the control. Exercised aged rats revealing downregulation of desmin (c), immunoreactivity with upregulation of VEGF (f), aquaporin-3 (i), and beclin-1 (l) immunoreactivity compared with the aged-S group. The inserts indicate a positive reaction (40x, scale bar = 20 *µ*m). *N* = number of rats.

**Figure 9:**
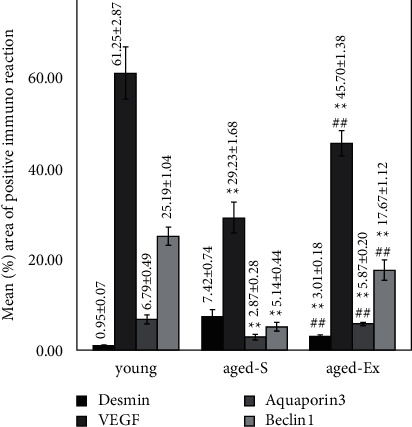
The histogram denoting the mean % area of positive desmin, VEGF, aquaporin-3, and beclin-1 immunoreactivity in the young, aged-S, and aged-Ex groups. ^∗∗^indicates *P* < 0.001 compared with the young group; ^##^indicates *P* < 0.001 compared with the aged-S group. The data expressed in mean ± SD (*n* = 10).

The renal tissues of the aged-S group showed significantly higher (*P* < 0.001) desmin immunoreactivity, indicating podocyte injury, than the young group. However, the aged-Ex group exhibited significantly decreased (*P* < 0.001) desmin immunoreactivity compared with that of the aged-S group. A significant decrease was noted (*P* < 0.001) in the VEGF, AQP3, and beclin-1 immunoreactivity within the renal tissue of the aged-S group compared with those of the control. On the contrary, treadmill exercise significantly increased (*P* < 0.001) VEGF, AQP3, and beclin-1 immunoreactivity in the aged-Ex group compared with those in the aged-S group. Furthermore, regarding the different immunoreactions, a significant difference was observed (*P* < 0.001) between the aged-Ex rats and the controls (Figures 8 and 9).

## 4. Discussion

Recently, the aging kidney became a topic of great interest in geriatric medicine and clinical nephrology [29]. This study states that treadmill exercise training may play a pivotal role in mitigating kidney aging. Moreover, to the best of our knowledge, this is the first study focusing on different mechanisms by which exercise affects the changes in the aged kidney.

Consistent with a previous report [30], aged-S rats had impaired renal function, reflecting the structural tubular changes and impairment of the glomerular filtration rate as postulated by Musso et al. [31]. This was in line with the results in this study, confirmed by the significant increase in tubular injury score in the aged-S group compared with that of the young group.

On the contrary, exercise training improved plasma urea and cystatin C levels; however, it had an insignificant effect on creatinine and creatinine clearance. Dharnidharka et al. [32] clarified that serum cystatin C is a better filtration marker than serum creatinine, which could be due to the influence of physical exercise and muscle mass on plasma and urinary creatinine but not cystatin C levels [33]. One potential explanation for the insignificant difference in creatinine and creatinine clearance between the aged-S and aged-Ex groups may be the probability of increased muscle mass in the exercised group and the worse kidney function in the sedentary rats. The significant reduction in body weight of aged-Ex rats compared with aged-S rats could be attributed to the improved renal function with exercise training and subsequently decreased fluid retention [34].

In this research, the impaired renal function in aged-S rats was consistent with histopathological alterations. The renal cortex was chosen for histopathological assessment because it is more affected by age than the medulla [35]. The reported histopathological changes in this study were in agreement with those in previous research [30, 36, 37].

In this study, these histological alterations were accompanied by an insignificant difference in kidney weight, which was in line with the study by Denic et al. [38]. They attributed these alterations to the combination of the age-related renal sinus fat increase and increased medullary volume with the decrease in cortical volume.

The histological changes in the aged kidney in this work were previously explained in different nephropathy models. The presence of periglomerular fibrosis, which disrupted glomerular outflow and led to cystic changes in Bowman's space, could explain the dilation of Bowman's space [39]. On the other hand, glomerular hypercellularity, as a compensatory mechanism, could have resulted in the absence or narrowing of some Bowman's spaces [40].

Moreover, increased cell membrane permeability led to cytoplasmic vacuolation [41]; pyknotic nuclei indicated apoptotic changes [42]. In addition, cast formation indicated tubular injury, as clarified by Rahman and Purwakanthi [43]. Partial tubular obstruction or alterations in the structure of the tubular basement membrane were responsible for tubular dilatation [44]. Furthermore, intense interstitial hemorrhage could be attributed to inflammation and vascular injury [45].

Glomerulosclerosis is considered one of the characteristic features of kidney aging [46]. It may be attributed to many factors such as small renal arteriosclerosis with subsequent ischemic injury to nephrons [38], oxidative stress [47], and hypertension [48]. In this study, glomerulosclerosis attributed to the presence of thick-walled blood vessels, increased systolic blood pressure, and elevation of oxidative markers.

In the current work, most histopathological changes were alleviated by exercise, which was in agreement with previous research examining distinct exercise modalities on different nephropathy models [49, 50]. On the contrary, Moningka et al. [51] reported that exercise did not effectively alter the age-induced chronic kidney changes in the aging Fisher 344 male rats. This discrepancy could be attributed to the use of different rat strain and exercise protocol.

The mechanisms by which treadmill exercise alleviated age-related kidney changes could be understood by investigating the pathophysiology of kidney aging. The pathogenesis of age-related kidney changes is still poorly clarified. Several clinical and experimental studies have shown that oxidative stress increases with normal aging, which was in line with our results [13, 52].

Moreover, this study proved that aging is associated with chronic low-grade systemic inflammation, agreeing with the results of a previous report [53]. Renal fibrosis occurred due to elevated levels of inflammatory cytokine [54] with a subsequent imbalance between production and degradation of extracellular matrix, epithelial-to-mesenchymal transition, and fibroblast activation [55]. In the present study, renal fibrosis was confirmed by the significantly increased percentage area of collagen deposition in the aged-S rats compared with that in young rats.

Furthermore, the favorable effects of exercise were attributed mostly to its antioxidant and anti-inflammatory nature. Both inactivity and high-intensity exercise increased oxidative stress, whereas moderate-intensity exercise reduces oxidative stress [56]. Therefore, moderate-intensity exercise was performed in this work.

The antioxidant effect of exercise was confirmed in this study. According to a previous study [57], exercise training significantly improved the oxidant/antioxidant imbalance in the aged rats. Sallam and Laher [58] indicated that the anti-inflammatory effect of exercise is attributed to its role in the modulation of anti-inflammatory/proinflammatory cytokine profiles. The effect of exercise on different inflammatory markers is controversial. In this study, exercise training reduced plasma CRP but not IL-6 levels in aged-Ex rats compared with those of aged-S rats, which could be because CRP is more responsive to physical activity than IL-6 [53]. Furthermore, in the present study, treadmill exercise alleviated the progression of renal fibrosis in the aged rats mostly due to its anti-inflammatory properties, which was in line with Huang et al. [59]. They noted that renal fibrosis was reduced with exercise in a hypertensive rat model.

Different mechanisms are also implicated in renal pathology in aged-S rats. Hence, studying the modulation of these mechanisms reflected the beneficial role of treadmill exercise on the aged kidney. The present study revealed that podocyte injury, indicated by upregulation of desmin, and downregulation of VEGF, autophagy, and AQP3 were implicated in aging-induced renal changes, which was in line with Poulaki et al. [60] and Durvasula and Shankland [61], who attributed the different aging-related renal changes to podocyte injury.

Moreover, Yamaji et al. [62] observed a reduction in the VEGF gene in the aged kidney. Low VEGF could be responsible for podocyte injury, glomerulosclerosis, and tubulointerstitial fibrosis development [63]. In addition, a low VEGF level is implicated in endothelial dysfunction with subsequent hypertension [64]. Peralta et al. [65] declared a strong association between kidney function and systolic blood pressure.

Now, the expression and modulation of AQPs are intensely investigated around the world [66]. In this study, the downregulation of AQP3 immunoreaction in aged-S rats was consistent with a previous report [67]. Preisser et al. [68] studied AQP1-4 expression in the aged kidney. They reported no change in the expression of AQP1 and AQP4 and found downregulation of AQP2 and AQP3. Indeed, Lei et al. [69] confirmed that AQP3 deletion provoked kidney injury by increased apoptosis. Moreover, Xie et al. [11] attributed the reduction in AQP3 expression to increased oxidative stress.

The impact of autophagy on aging and progression of kidney disease is still not fully understood [16]. Autophagy-related proteins are involved in the execution of autophagy. Beclin-1 participates in the early stages of autophagy. It promotes the nucleation of the autophagic vesicle and recruits proteins from the cytosol [70]. In this study, autophagy was downregulated in the aged kidneys, which was in line with Cui et al. [71], who observed downregulation of autophagy-related gene light chain 3 in the aged kidneys. The precise mechanisms leading to age-associated autophagy reduction remain unclear, which could be due to a general failure in maintaining autophagy-related protein expression [72].

In the current study, treadmill exercise modulated different mechanisms via protecting the podocytes from injury, improving VEGF and AQP3 expression, and inducing kidney autophagy. Agreeing with these results, Ishikawa et al. [73] attributed the beneficial effects of exercise in diabetic kidney disease to the maintenance of podocytes, with alleviation of oxidative damage and inflammation.

Furthermore, the improvement in systolic blood pressure in aged-Ex rats was in line with previous report [74], which could be explained partially by upregulation in VEGF. Pourheydar et al. [75] considered exercise an innovative nonpharmacological therapy that adjusted VEGF protein level in the aged heart and hypothesized that exercise improved angiogenesis. The exercise-induced VEGF upregulation and the subsequent alleviation of the age-induced microvascular deterioration could be attributed to the antioxidant mechanism of exercise, as postulated by Viboolvorakul and Patumraj [76] due to the repeated exposure to increases in blood flow and shear stress during exercise [77].

Indeed, the significant increase in AQP3 immunoreactivity was consistent with the results of Amer and Al-Sharaky [78] who noted that swimming exercise modulated AQP3 renal expression. The exact mechanism by which exercise improves the AQP3 expression in the aged kidney is not well understood. However, the improvement of AQP3 expression could be attributed to the antioxidant properties of exercise since the impairment of AQP3 in the aged kidney was related to the aging-associated oxidative stress. Further studies are warranted to investigate in-depth the exact mechanism.

Moreover, Brandt et al. [79] stated that exercise could induce autophagy and considered exercise a stress stimulus, modulating cellular signaling and promoting metabolic adaptation. Zhou et al. [80] suggested that podocyte autophagy is involved in renal protection and may be a therapeutic target. Aerobic exercise could improve autophagy due to its capability of inhibiting the phosphorylation of mammalian target of rapamycin by upregulating the activity of adenosine monophosphate-activated protein kinase, thus ameliorating aging-associated changes. [15].

## 5. Conclusions

The present study demonstrates that moderate-intensity treadmill exercise training improved structural and functional age-related changes in the kidney of aged rats. Thus, aerobic exercise is highly recommended for elderly people for protection against kidney aging. Further studies are warranted to investigate in-depth the different underlying mechanisms.

## Figures and Tables

**Table 1:** Weight (body and kidney) and biochemical parameters of the different groups.

Parameter		Young	Sedentary aged	Exercised aged
Weight	Body weight (g)	187 ± 10.0	430 ± 12.4^*∗∗*^	405 ± 10.0^*∗∗*^##
	Relative kidney weight (g/100 g body weight)	0.54 ± 0.03	0.52 ± 0.01	0.53 ± 0.02

Kidney function tests	Plasma creatinine (mg/dL)	0.72 ± 0.06	0.82 ± 0.06^*∗*^	0.77 ± 0.06
	Plasma urea (mg/dL)	40.2 ± 1.8	53.6 ± 5.2^*∗∗*^	45.3 ± 7.1#
	Plasma cystatin C (mg/L)	0.91 ± 0.15	2.5 ± 0.28^*∗∗*^	1.6 ± 0.42^*∗∗*^##
	Creatinine clearance (mL/min)	1.27 ± 0.10	0.94 ± 0.06^*∗∗*^	1.02 ± 0.5

Oxidative/antioxidative markers	MDA (nmoL/mL)	5.14 ± 0.99	11.39 ± 1.75^*∗∗*^	8.96 ± 1.60^*∗∗*^#
	TAC (nmoL/L)	2.49 ± 0.12	2.14 ± 0.17^*∗∗*^	2.44 ± 0.07##

Inflammatory markers	CRP (mg/L)	3.20 ± 0.78	8.00 ± 1.56^*∗∗*^	5.20 ± 0.78^*∗∗*^##
	IL-6 (pg/mL)	148 ± 12.2	161 ± 8.9^*∗*^	152 ± 11.0

Values are presented as mean ± SD (*n* = 10). ^∗^indicates *P* < 0.05; ^∗∗^indicates *P* < 0.001 compared with young group; ^#^indicates *P* < 0.05; ^##^indicates *P* < 0.001 compared with sedentary aged group. MDA: malondialdehyde. TAC: total antioxidant capacity. CRP: C-reactive protein. IL-6: interleukin-6.

## Data Availability

The data used to support the findings of this study are available from the corresponding author upon request.
